# The Touch of Madness:Manto as a Psychiatric Case Study

**DOI:** 10.12669/pjms.295.3882

**Published:** 2013

**Authors:** Ali M. Hashmi, Muhammad Awais Aftab

**Affiliations:** 1 Ali M. Hashmi, MD, Foreign Professor III (HEC), Psychiatry, King Edward Medical University/Mayo Hospital, Lahore, Pakistan.; 2 Muhammad Awais Aftab, MBBS, Department of Psychiatry, King Edward Medical University/Mayo Hospital, Lahore, Pakistan.

**Keywords:** Manto, Creativity, Anxiety, Alcohol abuse, Madness

## Abstract

Saadat Hasan ‘Manto’ is widely acknowledged as one of the greatest short story writers in the world. He died at the age of forty three from complications of alcoholism. All of his life, he suffered from symptoms of anxiety and depression and his alcohol abuse was intimately linked both to his mental distress as well as his creative genius.

This paper examines the life of Manto from a psychiatric perspective and the link between creativity and mental illness. We show how Manto’s particular family circumstances led to the development of restlessness and later anxiety in his life; how his substance abuse, especially alcohol abuse exacerbated this mental distress and how it eventually led to his death and how all of these factors were intimately linked to his creative genius and were the source of so many of his literary masterpieces.

***Methods: ***We reviewed seventy five short stories considered to be his best. Writings about Manto’s life including his own were reviewed to construct a picture of his life as well as find clues to his mental distress and alcohol abuse. A literature search for articles related to creativity and mental illness was conducted using Google Scholar containing the search terms ‘creativity and madness’ and ‘creativity and mental illness’ in the titles of the articles. References most relevant to our case study were identified.

***Conclusion: ***Manto suffered from symptoms of anxiety and depression which today would meet the diagnostic criteria for Alcohol Dependence and, in later life, Alcohol-induced Psychosis. Appropriate treatment may have prolonged his life although that may have come at the expense of his creativity.

## INTRODUCTION

“Men have called me mad; but the question is not yet settled, whether madness is or is not the loftiest intelligence — whether much that is glorious—whether all that is profound—does not spring from disease of thought—from moods of mind exalted at the expense of the general intellect.” ***Edgar Allan Poe.***

Creativity and madness are intimately linked in popular imagination. The archetype of the “mad genius” is familiar to all of us. The psychological afflictions of Wolfgang Mozart, Vincent Van Gogh, Virginia Woolf, Sylvia Plath, Ernest Hemingway and countless other artists are the subject of ceaseless interest. The theme continues to abound in literature and biographies of literary and artistic legends are often brushed up by ‘a process of myth making idealization’ ascribing to them the allure of neurosis.^1^ Psychiatrists have been in the forefront of exploring this association. 

Andreasen reports from her study that creative writers had a substantially higher rate of mental illness, predominantly mood disorders (with a tendency toward bipolar disorder).^2^ There is also a higher prevalence of affective disorder and creativity in the first-degree relatives of writers, indicating that these traits could be genetically mediated. These conclusions are typical of other medical literature that exists on this topic.^3^ A study of 291 famous men reported a higher prevalence of depressive disorders, alcoholism, and less reliably, psychosexual problems, in writers.^4^ A follow-up study of one hundred American and British writers confirmed this high prevalence of affective disorders and alcoholism.^5^

Some of the most rigorous work on this subject, spanning several decades has been done by Arnold M. Ludwig. He concludes that while not a prerequisite, a touch of madness could enhance creativity.^6^ His work on women writers shows that almost any type of sustained emotional distress could be associated with creative activity, provided it is not incapacitating. The relationship is complicated, though and familial, developmental and environment factors have to be taken into account.^7^

The current psychological view is that it is the ‘softer’ manifestations of mental disorders that are associated with heightened creativity because they share some biological-cognitive-personality features, such as cognitive disinhibition.^8^

In this paper, we discuss, in the background of this link between creativity and mental illness, the life and work of Saadat Hassan Manto, the famously controversial short story writer.

***Early Life and Development: ***Amongst artists of recent times Manto is definitely the most suitable for a psychological analysis. Even a superficial study of his life indicates that he spent his entire life battling inner demons. In the last years of his life, he even spent time in psychiatric hospitals (or ‘insane asylums’) for treatment of his alcohol addiction (and while in one such place, crafted his masterpiece, ‘Toba Tek Singh’).

It would be best to begin a study of Manto’s personality from an ‘internal’ perspective, dealing with the inner psychodynamic forces of a person’s mind. This point of view derives from Freud’s ideas of psychoanalysis. It holds that every act of a person’s life, every thought, every emotion, is under the influence of unconscious forces which a person understands only to a very limited extent. According to this theory, the emotions and experiences that arise in a person’s mind from birth up to the age of four or five years form the basis of his unconscious mind, and later these factors are the source of every thought, emotion and action. Accordingly, understanding these unconscious factors is crucial in studying a person’s life.^9^

Manto was born in 1912 at Samrala, near Ludhiana. His father, Maulvi Ghulam Hussain, married twice. Manto’s mother was his second wife. Ghulam Hussain’s family was not happy with his second marriage; he constantly belittled Manto and this was the first source of bitterness and anger in the young Saadat’s life.^10^ Manto’s relationship to his father was based on respect and fear. Manto’s biographers say that this lack of fatherly affection was compensated for by his mother, Bibi Jaan.

Saadat’s relationship with his father is important. Freud’s theory of the Oedipus Complex^11^ states that a young child (Freud is referring here to the age group of 3 to 6 years) is both afraid of, and idealizes his father. The basic reason is the child’s observation that this man who is his ‘father’ is the companion of the person who is the child’s creator and his first and deepest love, i.e. his mother. According to Freud, the mutual love between the father and mother evokes feelings of anger and jealousy which are repressed for the satisfaction of his parents. These emotions provide the basis of his unconscious.^11^

Examining Manto’s stories from this perspective, the reader repeatedly finds that the male characters in Manto’s celebrated short stories are often of secondary importance. Manto’s woman, whether a prostitute or a homemaker, possesses more courage and perseverance than his male characters. Where male characters are prominent in his stories, it is primarily for their barbarity and cruelty – perhaps a reflection of Manto’s fear of his father. Not that Manto’s women are paragons of affection and purity, but in his best short stories, Manto presents his female characters with great sympathy and love. From a psychological point of view we can say that this is a reflection of his feelings of love and admiration for his mother, an extension of the Oedipal relationship.

By 1930, when his father died, Manto had failed twice in his FA examination and was fed up with education. In addition, his natural restlessness, the source of his creativity as well as later his alcoholism, was growing. He wrote: ‘In those days of vagrancy, I felt constantly dissatisfied. A strange restlessness gripped my heart and mind. (I) smoked hash with friends, took cocaine, drank (alcohol), but that disquietude would not go away.’^12^ (Lest we dismiss this description as the ‘existential angst’ of a maladjusted adolescent, he describes similar sentiments repeatedly throughout his life.)

***Association between Mental Illness and Substance Abuse: ***There is a strong association between alcoholism and other psychiatric conditions. Swendsen et al examined the comorbidity of alcoholism with anxiety and depressive disorders in four epidemiologic investigations from diverse geographic sites. They reported that individuals with alcohol abuse or dependence generally experienced a twofold to threefold increased risk of anxiety and depressive disorders. While the presence of comorbid anxiety or depressive disorders was consistently associated with moderate increases in the symptoms of alcohol abuse or dependence, alcoholism was associated with large increases in the number of depressive symptoms.^13^ It should be remembered here that alcoholism itself is a serious psychiatric disorder, with the DSM-IV containing diagnoses of both Alcohol Abuse and Alcohol Dependence.

Tómasson and Vaglum reported that over 70% of pure alcoholics and over 90% of polysubstance users had co morbid diagnoses. The most prevalent disorders were: affective (33%), anxiety (65%), antisocial personality disorder (28%) and psychosexual dysfunction (20%).^14^

It is also relevant here to bring up the “self-medication hypothesis”^15^ which states that drugs alleviate or alter psychological suffering and by utilizing these drugs, the patient is essentially self-medicating and becoming an addict in the process. Khantzian states that patients with substance use disorders experience extremes of emotions. They are often either overwhelmed with painful states of mood or they do not appear to feel their emotions at all. Substances of abuse assist such individuals to allay painful affective states or to experience or control emotions when they are absent or confusing.^16^ From a psychodynamic perspective, the drug's effects substitute for defective or non-existent ego mechanisms of defense. The choice of the substance of abuse is therefore not arbitrary. A person might experiment with a variety of drugs, but his ultimate drug of choice is the one which modulates their specific type of psychological distress. The addict's preference for a particular drug is a result of the interaction between the psychopharmacologic properties of the drug and the psychological states of distress from which the addict desires respite.^17^

Manto was plagued by a constant restlessness and unease in life, which may very well be consistent with a clinical diagnosis of anxiety and depression. As one would expect from the self-medication hypothesis, Manto sought refuge in substance abuse to ease his psychological pain. In his case, the drug of choice was alcohol. Given the non-availability of Manto’s psychiatric treatment records and the retrospective nature of this analysis, we can only speculate as to the validity of the diagnosis of depressive disorder and/or anxiety disorder. No psychiatric diagnosis can or should be made retrospectively, especially without access to the subject or any documentation. However, considering the co-morbid associations of alcoholism and evidence from Manto’s life, we believe it to be a likely possibility.

***The Origins of Manto’s Distress: ***Where did this restlessness in Manto come from? In the milieu of 20^th^ century existential philosophy, Manto’s writing also reveal the existential conflict which flows from the individual's confrontation with the “givens of existence”.^18^ These are ultimate human concerns, such as the awareness of the inevitability of death, our “terrifying freedom to choose”, our existential isolation and the apparent meaninglessness of the world.^18^Manto as a sensitive soul was acutely aware of these concerns and this awareness took a toll on his psyche.

In his writings though, there is no hint of Manto’s mental and spiritual agitation. Manto’s literary eye was like a camera which observed the events happening to his characters without blinking and recorded them as they were. Consider this small excerpt, for instance:“At fifteen past seven, the police took away the dead body. Ice and blood remained behind on the road. A tonga passed by. The child saw the shiny clot of frozen blood on the road. His mouth watered. He tugged his mother’s arm and pointed at it. ‘Look, mummy, jelly.’ [‘Jelly’].^19^

Manto was deeply affected by the horrible events he witnessed during the partition of the subcontinent. He expressed his agitation several times in his works:“If you are unaware of the times through which we are passing, read my short stories. If you cannot tolerate them, it means this age is intolerable. There is no fault in my writing. The fault which is attributed to my name is actually the fault of the current system. I do not want to agitate people’s thoughts and emotions. How can I disrobe civilization, culture and society when it is in fact already naked?”.^20^

Although Manto’s life in Bombay was relatively prosperous, that inner restlessness and Bombay’s particular social environment (in which his relationship with the Bombay film industry is prominent) led to indiscriminate drinking. He was slowly becoming an alcoholic and the coming years were only to aggravate it. In 1947 Manto decided to migrate to Pakistan. Moving to Lahore was not a fortunate development. Pakistan’s film industry was desolate due to the migration of non-Muslim artists. No studio was functional. Manto had no way to earn a living in Lahore. In his last years his financial troubles escalated along with his restlessness and distress. Often he would go to a newspaper office, write a short story while sitting there, and buy a bottle of alcohol with the money received. The newspaper staff knew of this habit, so they would sometimes hand him a cheap bottle of alcohol instead of money. He was admitted to hospital several times.

Furthermore, Manto faced several trials on charges of obscenity. Even though he was honorably released from all these trials, the legal proceedings would have been trying for a man with no employment and economic hardship.

His drinking escalated. Signs of madness began to appear. He hallucinated, saw ghostly faces and talked nonsense. Alcohol induced psychosis is a recognized clinical entity and is associated with poor outcomes.^21^

His wife Safia tried to get him treated several times and even got him admitted to a mental hospital (after which he wrote his great story, ‘TobaTek Singh’), but all in vain. He died from inflammation of the liver related to his drinking soon after. He wrote, in a letter to Ahmed Nadeem Qasmi, in January 1939:‘Whatever happens, I can find no satisfaction. I am not satisfied with anything. I am not even satisfied with myself. I feel as if who I am, what is inside me, should not be so. It should have been different.’^12^

Despite all these problems, in his seven years in Lahore, Manto wrote 127 short stories as well as two collections of essays, two collections of sketches, a collection of the account of his trials, and a novelette, Beghair Unwaan Kay (Untitled). This shows that despite growing mental and physical weakness, Manto’s creative faculties remained intact till his last breath, though some of his last stories (for instance, ‘Phundnay’ /Tassels) give the impression of being the work of a madman.

***Creativity and Madness: ***Ludwig writes that the relationship between creativity and madness can be very divergent. For some writers, writing can be a therapeutic exercise while for others it is the creative process itself that brings out their inner demons. One can see these dual mechanisms at play in Manto. Writing was therapeutic for him, but the gravity of what he wrote about had an emotional cost of its own.^7^

Given the lack of definitive evidence, no direct confirmation can be provided to show that Manto’s creativity was indeed fuelled by his alcoholism and possible co-morbid psychiatric conditions. However, it remains a possibility that this was, indeed the case. As mentioned before though, without corroborative evidence, a ‘diagnosis’ cannot (and should not) be made.

Evidence tends to support an inverted-U relationship between mental illness and creativity, such that creativity tends to increase with milder forms of mental illness but decreases as the disorder becomes more severe and functionally disabling. This relationship has been shown for affective disorders (depression and bipolar disorder) as well as the schizophrenia spectrum. Dysthymic, Hypomanic and Schizotypal traits are related to artistic creativity, but it declines with severe forms of these conditions such frank Mania and Schizophrenia.^22^

We see this pattern in Manto’s life as well. His psychological agitation and alcohol abuse promoted his creativity, but once these underlying problems became severe enough to be incapacitating, there was a sharp decline.

Manto’s life circumstances aggravated his natural restlessness. His treated this by drinking and this addiction finally took his life. In his last few years he knew very well that alcohol was poison for him yet he did not stop.

He wrote “You would not believe, uncle, that despite being the author of twenty, twenty two books, I do not own a house to live. If an essay of mine gets published in the newspaper, and if I earn twenty, twenty five rupees based on the rate of seven rupees per column, I take the tonga and go buy locally distilled whiskey. Had this whiskey been distilled in your country, you would have destroyed the distillery with an atom bomb, because in just one year this stuff is guaranteed to send a man to kingdom come.”(‘Chacha Sam keNaamKhat’ / First Letter to Uncle Sam).^23^

***Eros and Thanatos: ***The passage above shows not only the destructive effects of alcoholism on a person’s life but also Manto’s awareness of this self-destruction. He continued drinking, as if he consciously chose death, like a man who shoots himself in the temple or jumps in front of a train. In psychological terminology such behavior is termed as the Death Drive. This is the force, according to Freud, which is active within all life and is forever pushing it towards annihilation. Freud called this force Thanatos, the personification of death in Greek mythology. In contrast, Eros is the force of life, which drives life towards growth and sustainment.^24^

According to Freudian psychology, the influence of continuous external trauma on Manto’s natural and inborn psychological sensitivity eventually forced him to surrender to death. Some psychologists may even say that Manto’s burst of creative output in Lahore (despite the increasing poverty and ill health) was a sign that he was not only unconsciously aware of his impending death but was in fact preparing for it.

“There is no great genius without some touch of madness,” wrote Seneca. Manto was such a genius, and he paid a dear price for it.

## Author contributions:

***Ali Madeeh Hashmi*** was involved in conception of the study, literature review, manuscript writing and final approval. ***Muhammad Awais Aftab*** contributed to outline of the study, literature search and review, drafting the article and final revision.
